# Grey and harbor seals in France (mainland and Saint-Pierre et Miquelon): microbial communities and identification of a microbial source tracking seal marker

**DOI:** 10.3389/fmicb.2024.1484094

**Published:** 2024-12-04

**Authors:** Alisson Godino Sanchez, Joëlle Serghine, Cécile Le Mennec, Cyril Noël, Julien Schaeffer, Herlé Goraguer, Cécile Vincent, Thomas Vitré, Françoise S. Le Guyader, Michèle Gourmelon

**Affiliations:** ^1^IFREMER, ODE-DYNECO-PELAGOS, Plouzané, France; ^2^IFREMER, U. Microbiologie Aliment Santé et Environnement, LSEM, Nantes, France; ^3^IFREMER, IRSI, SeBiMER Service de Bioinformatique de l'Ifremer, Plouzané, France; ^4^IFREMER, Délégation Saint-Pierre et Miquelon, Saint-Pierre et Miquelon, France; ^5^CEBC UMR7372 CNRS-La Rochelle Université, La Rochelle, France

**Keywords:** Grey seal, harbor seal, bacterial communities, fecal contamination, microbial source tracking, RNA virome

## Abstract

**Introduction:**

Seals, protected wild marine mammals, are widely found in waters around the world. However, rising concerns about their increasing numbers in some areas have led to potential worries regarding microbiological contamination of coastal areas by their feces, which could impact bathing and shellfish-harvesting activities. To the best of our knowledge, no study has been conducted on the bacterial and RNA viral communities present in the feces of both grey and harbor seals, which are the two main seal species observed in mainland France and overseas.

**Methods:**

Fecal bacterial (*n* = 132) and RNA viral (*n* = 40) communities of seals were analyzed using 16S rRNA gene amplicon high-throughput sequencing and viral RNA sequencing methods, respectively. In addition, to identify the specific characteristics of seal fecal microbial communities compared to other animal fecal microbial communities that may also contaminate coastal areas, the bacterial communities of seals were compared to those of wild waterbirds and breeding animals (i.e., cattle and pigs) which could be present in upstream catchments of coastal areas. Finally, ANCOM was used to identify unique and seal-associated Amplicon Sequence Variants (ASVs), aiming to develop a Microbial Source Tracking (MST) bacterial qPCR marker associated with seals.

**Results and discussion:**

The bacterial communities of grey and harbor seals were not found to be significantly different and were characterized by a predominance of Firmicutes, including the genera *Clostridium sensu stricto* 1 and *Peptoclostridium*, followed by Fusobacteriota with the genus *Fusobacterium*, and Bacteroidota with the genus *Bacteroides*. However, variations in bacterial communities between sites and individuals were observed. Similar observations were made for the RNA viral communities being characterized by a predominance of *Picobirnaviridae* (44% of total reads) and *Astroviridae* (15%). This study successfully developed a sensitive (89.8%) and specific (97.1%) MST qPCR marker targeting grey seal-associated bacteria belonging to the Bifidobacteriaceae family. This marker can be used to identify potential fecal contamination of coastal areas by seals and complements the MST toolboxes of markers already developed for humans, wild birds and livestock.

## 1 Introduction

**Figure 1:**
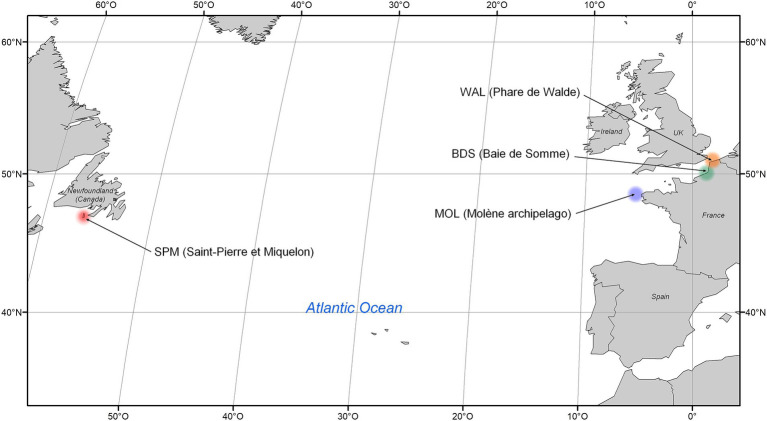
Location of seal feces sampling sites.

Marine mammals such as pinnipeds, diving marine predators, play crucial ecological roles in the oceans, but little is known about their microbiotas (Bik et al., 2016; Numberger et al., 2016; Pacheco-Sandoval et al., 2019). The present study focused on the two most frequent seal species in France along the English Channel coast and in Saint-Pierre et Miquelon overseas (Figure 1): i.e. grey seals (*Halichoerus grypus*) and harbor seals, also named common seals (*Phoca vitulina*) (den Heyer et al., 2021; Poncet et al., 2021; Savouré-Soubelet et al., 2016).

Grey and harbor seals in mainland France are at the southern limit of the species’ ranges in the North-eastern Atlantic (Vincent et al., 2017). In Saint-Pierre et Miquelon, both species occur in smaller numbers that in the adjacent Canadian waters, although recent and frequent abundance estimates are only available for the grey seal (den Heyer et al., 2021). Most recent counts on haulout sites gave an annual maximum of 2,635 grey seals and 1,319 harbor seals in mainland France, and 218 grey seals and 1,652 harbor seals in Saint-Pierre et Miquelon, in 2021 (Poncet et al., 2021; Poncet et al., 2023). Both these seal species are protected at the international and European level (Appendix III of the Bern Convention and Annex II of the Bonn Convention EEC, 1992).

Grey and harbor seals are considered opportunistic predators, top-tier consumers that feed on benthic or/and pelagic species, mainly fish and cephalopods (Pacheco-Sandoval et al., 2019; Planque et al., 2021). They are sympatric in the North Atlantic and can share the same haul-out sites as well as potentially similar foraging grounds (Planque et al., 2021). Furthermore, they move and feed within short distances of the coast, i.e., few tens of kilometers from the shore for harbor seals and few hundreds of kilometers for some grey seal individuals (Cunningham et al., 2009; Härkönen et al., 1999; Harvey et al., 2008; Thompson et al., 1996; Vincent et al., 2017). The diet of seals could be different according to species and location. Seal feces analyzed in this study came from four different locations: i.e. Baie de Somme (BDS), Walde (WAL), Molène archipelago (MOL; Ile de Morgol), and Saint-Pierre et Miquelon (SPM; Le grand barachois, Miquelon).

In the Baie de Somme and Walde, grey seals feed predominantly on flatfish (61%), including Pleuronectidae (plaice and flounder) and Soleidae (mainly common sole and sand sole), and, to a lesser extent, on herring (18%) and Gadidae, such as whiting and poulting (Planque et al., 2018). Their diet also includes cephalopods, such as squid, and Chondrichtyes (rays or ground sharks, found in 3.2% of the samples). Harbor seals mainly feed on flatfish (86%): common sole, sand sole, yellow sole, thickback sole, plaice and flounder, and, to a lesser extent, on common dragonet (6%) (Planque et al., 2018). In the Iroise Sea (i.e., Molene archipelago), grey seals prefer ballan wrasse and the other round fish such as conger, pollack, sea bass or pout (Ridoux et al., 2007). Finally, the diet of grey and harbor seals in Saint-Pierre et Miquelon is currently being studied. Preliminary results suggest grey seal mainly feed on sandeels (Ammodytidae) and cod, but may also consume crustaceans, while harbor seals mainly feed on sticklebacks (Gasterosteidae), although this diet estimate may be biased seasonally (Vincent et al., 2022).

Seals, mainly piscivorous, have a diet high in proteins and polyunsaturated fatty acids, different from diet of other animals, such as terrestrial carnivorous mammals (Ley et al., 2008; Nelson et al., 2013). When they compared the fecal bacterial community of a variety of terrestrial mammals, Ley et al. (2008) identified that diet in addition to host phylogeny and, to a lesser extent, gut morphology influenced the composition of the gut bacterial community.

Due to their high trophic level diet but also to their long life, wide distribution, coastal habitat and possible infection with bacteria, viruses, and parasites that could also be pathogens for humans, marine mammals such as seals can serve as sentinel species for coastal ecosystem health (Hughes et al., 2013; Baily et al., 2015; Simeone et al., 2015). Indeed, the presence of bacterial, viral, and fungal agents of marine mammal zoonoses has been reported in seals: among the bacteria, e.g., *Brucella* spp., *Clostridium* spp., *Erysipelotrix* spp., *Mycobacterium* spp., *Mycoplasma* spp., *Photobacterium* spp., *Vibrio* spp. (reviewed in Waltzek et al., 2012; Hughes et al., 2013; Siebert et al., 2017; Pacheco-Sandoval et al., 2024). In addition, pathogenic bacteria, that can cause food poisoning in humans, such as *Campylobacter* spp., *Salmonella* spp. and *Clostridium perfringens*, have also been isolated from seal feces (Nelson et al., 2013; Greig et al., 2014; Lisle et al., 2004; Baily et al., 2015; Stoddard et al., 2007). In addition, fecal indicator bacteria (FIB) such as *Escherichia coli* which are used to highlight a fecal contamination in bathing and shellfish-harvesting areas, have been found to be present in seal feces, as they are in the feces of other marine and terrestrial mammals (Calambokidis and McLaughlin, 1987).

More globally, the microbial communities present in seal feces have been the subject of little research (Numberger et al., 2016; Pacheco-Sandoval et al., 2019; Switzer et al., 2023; Pacheco-Sandoval et al., 2024). They have been shown to differ from those of terrestrial mammals and humans, with, e.g., a significantly higher average relative abundance of a phylum such as Fusobacteriodota (Nelson et al., 2013; Switzer et al., 2023).

Questions may also be raised about viruses shed by these animals. The marine environment is known for its huge diversity of viruses (Sunagawa et al., 2020) and the potential emergence of human pathogenic viruses has been raised mainly for RNA viruses belonging to the *Picornaviridae* or *Caliciviridae* families (Bergh et al., 1989). Recently, few studies have been conducted in fur seals to describe the virome, either in feces (Kluge et al., 2016; Martínez-Puchol et al., 2022), or from spleen and lung samples (Canova et al., 2021). DNA viruses, such as circovirus and adenovirus, have also been described in fur seals feces (Chiappetta et al., 2017). In addition to human contamination, such as wastewater treatment plant discharges and livestock effluents, wildlife can potentially introduce fecal contamination into coastal waters (Boukerb et al., 2021; Rince et al., 2018). Indeed, a high diversity of viral sequences was detected in shellfish samples in a wildlife-contaminated area (Bonny et al., 2021). One possible consequence is the negative impact on human health through shellfish consumption or water ingestion, and the increased risk of zoonotic transmission through contaminated shellfish (Zakhour et al., 2010). The ability to describe the different communities may help to prevent zoonotic events in a One Health approach (Santiago-Rodriguez and Hollister, 2023).

The microbiological quality of coastal waters can be affected by fecal material originated from anthropogenic sources (e.g., wastewater treatment plant effluent, agricultural run-off) and from wildlife sources (e.g., wild bird droppings and the feces of marine mammals such as seals). In fact, seal haul-out sites can result in large amounts of feces being deposited in coastal waters, such as recreational areas, which can lead to poor water quality and beach closures (Paar et al., 2024). Microbial Source Tracking (MST) is a discipline which uses host-associated (e.g., bacterial, viral or chemical) markers to discriminate between human and animal excreta and to distinguish animal hosts as an approach to identify, and ultimately remediate sources of fecal contamination (Harwood et al., 2014). Tested and validated for source specificity and sensitivity, MST markers can be used as an MST toolbox for routine water quality monitoring in coastal environments (Gourmelon et al., 2010; Jarde et al., 2018; Sinigalliano et al., 2021). The MST markers were essentially based on qPCR bacterial markers associated with different hosts, but other markers developed using, for example, RNA viral sequencing could also be useful tools for tracing sources of fecal contamination (Sinigalliano et al., 2021; Santiago-Rodriguez and Hollister, 2023). Few microbial MST markers associated with wildlife have been developed and a seal-associated microbial marker to assess potential seal contamination of coastal waters is still lacking (Paar et al., 2024).

The main objectives of this study were the identification of microbial (i.e., bacterial and viral) communities in the feces of the two most frequent seal species (grey seal: *Halicheorus grypus* and harbor seal: *Phoca vitulina*) in France (Saint-Pierre et Miquelon island and mainland France) using 16S rRNA gene amplicon high-throughput sequencing and viral RNA sequencing and the investigation of MST microbial markers associated with seal fecal contamination.

## 2 Materials and methods

### 2.1 Fecal sample collection and location

Fresh fecal samples from seals [96 grey seals, 29 harbor seals and seven unidentified seals for which species was not certain, *n* = 132] were collected from 2015 to 2020 in mainland France as well as in Saint-Pierre et Miquelon (SPM) (Table 1). Individual feces were collected with a sterile spatula, retaining the inner part and ensuring no cross-contamination from the environment. The samples were either frozen at −20°C on site [e.g. SPM and Bay de Somme (BDS) samples] or sent on dry ice within 2 days to the laboratory where they were then frozen at −80°C until use. All samples were aseptically homogenized.

**Table 1:** Description of seal fecal samples (*n* = 132).

	Mainland France			Saint-Pierre et Miquelon
Sites	BDS	WAL	MOL	SPM
Grey seals	32	11	20 (20)*	33 (8)*
Harbor seals	15	0	0	14 (12)*
Not specified species seals	0	0	0	7 ^++^ (2)*
Total number of seal feces	47	11	20	54
Date of collection (years)	2017–2019	2017	2020	2015–2020

*() number of seal fecal samples analyzed for viral communities. ++Seal fecal samples not analyzed for bacterial communities.BDS, Baie de Somme; WAL, Walde; MOL, Molène archipelago; SPM, Saint-Pierre et Miquelon; Le grand barachois, Miquelon.

In addition, fecal samples collected from other sources [wild waterbirds, livestock (i.e., pigs and cattle); *n* = 322] from mainland France and described in a previous study (Boukerb et al., 2021) were used to compare bacterial communities in the seal samples described above to those of other sources.

### 2.2 Fecal sample treatments for RNA viral analysis

Fecal samples were thawed and mixed, with 1 g (wet weight) of the sample diluted in 9 mL of PBS. The mixture was vortexed at high speed for 30 s and then mixed vigorously for 20 s, repeated three times using a Fast-Prep apparatus. Sodium pyrophosphate was added to a final concentration of 10 mM, and the mixture was incubated for 40 min with gentle agitation at room temperature. This was followed by 3 cycles of sonication for 1 min each at maximum intensity using a cup-horn adaptor (Bandelin, HD 2200), with 1 min of cooling on ice in between. After centrifugation at 8,000 g for 20 min, the supernatants were collected, the pH was adjusted to 7 using HCl, and then 5 mL of 12% polyethylene glycol (PEG 60000) (Sigma-Aldrich, St-Quentin, France) was added. Following overnight incubation at 4°C with gentle agitation, the mixture was centrifuged at 13,000 g for 20 min at 4°C. The resulting pellet was resuspended in 2 mL of 0.05 M glycine buffer (pH 9) (Strubbia et al., 2019).

One negative control was prepared using 10 mL of the PBS used for fecal dilution, with the same processing steps applied as to the fecal samples. A positive control was made by inoculating 1 g of seal feces with an RNA virus, specifically norovirus GII.17 [P17], at a final concentration of 10^7^ genome copies/g. This positive control underwent all extraction and library preparation steps in the same manner as the other samples.

### 2.3 Total DNA extraction

Aliquots of approximately 0.25 g (wet weight) of seal feces were used for direct extraction of total genomic DNA using the FASTDNA™ Spin Kit for Soil (MP Biomedicals, Illkirch, France) as recommended by the manufacturer’s protocol and prepared under the conditions detailed by Boukerb et al. (2021). DNA was eluted in 100 μL sterile DNA/RNA free water. DNA quality and concentrations were assessed using a spectrophotometer (Epoch, BioTek) and a Qubit 4 fluorometer (Thermo Fisher Scientific). All DNA extracts were preserved at −80°C until the 16S rRNA gene amplicon library preparation.

### 2.4 RNA viral purification steps and nucleic acid extraction

The resuspended pellets were filtered through a series of filters with pore sizes of 5 μm, 1.2 μm, and 0.45 μm (Minisart NML 17594, NML17593, PES16533). The filtrates containing viral particles were recovered and free DNA and RNA were degrading by adding 2,000 units of OmniCleave Endonuclease (Lucigen, Wisconsin, United States) and the mixture was incubated for 1 h at 37°C. Nucleic acids (NA) were then extracted by adding 10 mL of lysis buffer (bioMérieux, Lyon, France) and 50 μL of paramagnetic beads (NucliSens kit, bioMérieux) (Strubbia et al., 2020). After performing washing steps using the eGENE-UP® apparatus (bioMérieux) according to the manufacturer’s instructions, the extracted NA were treated with 25 U TURBO™ DNase (Ambion, Thermo Fisher Scientific, France) for 30 min at 37°C. An additional RNA purification step was carried out using the RNA Clean & Concentrator™ -5 kit (Zymo Research, Irvine, USA) to remove DNase and PCR inhibitors.

### 2.5 16S rRNA gene library generation and MiSeq sequencing

The libraries were prepared (343F/784R, targeting V3-V4 hypervariable region of the 16S rRNA gene; Andersson et al., 2008; Liu et al., 2008) as detailed in Boukerb et al. (2021) and sequenced in three runs using the 2 × 250 paired-end method on an Illumina MiSeq instrument: (i) by the GeT-PlaGe platform (Toulouse, France) for the seal feces samples FPh1 to FPh20 and for the fecal samples from other sources [pig, bovine, poultry, and wild birds; described in Boukerb et al. (2021); Supplementary Tables S1A,B; May and November 2017], with a MiSeq Reagent Kit V3 chemistry (Illumina) and (ii) by the Bacterial Communication and Anti-infectious Strategies laboratory facility (Rouen, France) for samples FPh21 to FPh129 (Supplementary Table S1A; run performed 20 May 2021) with a MiSeq Reagent Kit V2 chemistry (Illumina).

### 2.6 Library and sequencing for viral analysis

All RNA extracts were converted to cDNA in triplicate using Superscript II (Invitrogen, Saint-Aubin, France) and random hexamers (New England Biolabs (NEB), United States) (Bonny et al., 2021). Negative and positive controls were transcribed only once. To minimize bias during library preparation, samples were processed in batches rather than individually. After double-strand DNA synthesis with the second strand reaction buffer from NEBNext Ultra RNA Library Prep, DNA fragmentation was carried out in 68 tubes using sonication (Ultrasonicator Covaris M220, duty factor: 5%, peak power: 75, cycles per burst: 200 for 195 s, Woburn, MA). Libraries were prepared using the KAPA Hyper Prep kit (Roche), following the manufacturer’s protocol. Each run included a negative control with sterile RNase-free water. The 128 libraries were sequenced in two separate runs using Illumina NextSeq 500 technology to generate 2×150 bp reads (iGenSeq, ICM, Paris, France).

### 2.7 Data availability

The 16S rRNA gene and RNA viral datasets generated for this study can be found in the European Nucleotide Archive (ENA) database (Study: ERP149173; Samples from ERS15965056 to ERS15970887 and from ERS16078207 to ERS16078302; details in the Supplementary Table S1C).

### 2.8 Bioinformatic analysis

#### 2.8.1 Bacterial communities

Raw data were analyzed using the SAMBA v4 workflow^1^ a Standardized and Automatized MetaBarcoding Analysis workflow using DADA2 (Callahan et al., 2016) and QIIME 2 (Bolyen et al., 2019) with default parameters unless otherwise indicated. Two SAMBA runs were performed, one including all seal fecal samples (*n* = 132) in addition to eight DNA extraction controls and one qPCR control (used at the microDecon step McKnight et al., 2019) and a second one including a selection of seal samples (i.e., only mainland France seal samples) and fecal samples from other sources also collected in mainland France (described in Boukerb et al., 2021).

This pipeline, developed by SeBiMER (Ifremer’s Bioinformatics Core Facility), is an open-source, modular NextFlow workflow (v22.10.4) (Di Tommaso et al., 2017) designed to process eDNA metabarcoding data. SAMBA is structured around three main parts: data integrity checking, bioinformatics processing, and statistical analysis. Firstly, the SAMBA verification step checked the integrity of the raw data. Then, sequencing primers were removed from the reads using Cutadapt (Martin, 2011) via QIIME 2 (v2022.11) (Bolyen et al., 2019), and reads without detected primers were discarded. Subsequently, the DADA2 package (v3.6.1) (Callahan et al., 2016) was used to filter out low-quality reads, correct sequencing errors, merge paired reads, infer Amplicon Sequence Variants (ASVs), and eliminate chimeras. Given the known issue of DADA2’s tendency to overestimate diversity, an additional ASV clustering step (OTU calling) was performed using the dbOTU3 algorithm (Olesen et al., 2017). Taxonomic classification was carried out using a naïve Bayesian classification against the SILVA database 138 (Glöckner et al., 2017; Quast et al., 2013), which was pre-filtered for the sequenced region (V3-V4).

Finally, SAMBA performed extensive analyses of the alpha- and beta-diversity using homemade R scripts (R Core Team, 2022) and ‘vegan’ package in R v4.0.2. Differences in alpha diversity indices among different fecal samples from different seal species and seal fecal samples from different sites were analyzed using Adonis statistical test and pairwise *t*-test.

ASV abundances for each sample were calculated at the phylum, family, and genus taxonomic levels. Alpha diversity within bacterial communities was characterized using the observed species richness and Shannon indices. Beta diversity was assessed through ordination analyses, specifically non-metric multidimensional scaling (NMDS) based on a weighted UniFrac distance matrix, with data normalized via Cumulative Sum Scaling (CSS) (Lozupone et al., 2011). To evaluate significant variance differences across indices by group or species, a permutational multivariate analysis of variance (PERMANOVA) was performed, followed by post-hoc pairwise comparisons.

To visualize the number of total, exclusive, and shared ASVs and genera among seal species, UpSet plots were created. Similarly, to Pacheco-Sandoval et al. (2024), to analyze the core group across species (i.e., grey seal and harbor seal), ASVs with a prevalence of 80% or higher in grey seal and harbor seal samples were considered, respectively, without setting a minimum relative abundance threshold.

#### 2.8.2 Viral communities

Bioinformatic analysis was conducted using a Nextflow pipeline as outlined by Bonny et al. (2021). For each sample, reads from the three libraries were combined for subsequent analysis (Schaeffer et al., 2023). Briefly, Fastq files were trimmed with fastp using a quality threshold of 25. Clean reads were deduplicated (CD-hit) and mapped to remove RNA contaminants (Silva RNA database) and PCR duplicates. *De novo* assembly was performed using metaSPAdes with k-mer lengths of 21, 33, 55, 77, and 99 (Nurk et al., 2017). Contigs longer than 300 bp were filtered and identified using BLASTn and BLASTx with an E-value threshold of 10^−5^ and nr bank using diamond with an e-value of 10^−3^. When both methods (i.e., BLASTn and BLASTx) provided results, the BLASTn assignment is retained. To assess contig coverage, post-processed reads were mapped to the metaSPAdes contigs using Bowtie2 (v2.3.0) (Langmead and Salzberg, 2012; Nurk et al., 2017). Multi-mapped reads were excluded to avoid potential overestimation of abundance. Only merged reads were considered for each sample after this step.

Taxonomic identification was performed using the Entrez Direct tool, which enabled extraction of information at specified taxonomic levels. A heatmap was generated using R. Reads per million (rpm) were calculated by dividing the number of reads per family by the total number of trimmed and deduplicated reads.

### 2.9 Identification of potential seal bacterial MST markers

Differences in the mean abundance of microbial taxa in seal feces compared to other fecal sources (e.g., pigs, cattle, and wild waterbirds) (Boukerb et al., 2021) were identified using ANCOM (Analysis of Composition of Microbiomes). In ANCOM analysis, the W value represents the number of times a particular ASV is found to be significantly different in terms of abundance in pairwise log-ratio comparisons with all other ASVs (Mandal et al., 2015).

Among the 83 unique and seal-associated ASVs identified by ANCOM, the sequences of the 20 ASVs with the higher W value were retained and subsequently compared to the sequences of the NCBI nucleotide database,^2^ including, e.g., other marine mammals such as dolphin sequences (Bik et al., 2016) and other animals, to identify, for each ASV, the top 20 matches with the greatest host diversity (seals and other non-targeted animals). These sequences were then aligned using BioEdit software with ClustalW Multiple Alignment to identify variable regions (specific to the target sequence) and conserved regions (shared with non-target sequences).

When seal-specific regions were identified, primers were drawn manually or using Primer3,^3^ OligoCalc (v3.27) (Kibbe, 2007) and Multiple Primer Analyzer (ThermoFisher).

### 2.10 qPCR assays

Pairs of primers were designed to target the four ASVs that were most specific for seals out of the 20 ASVs selected. One of these four ASVs was from *Fournierella* genus (Firmicutes), one from *Atopobium* genus (Actinobacteriodota), one from *Slackia* genus (Actinobacteriodota), and the last from the family Bifidobacteriaceae (Actinobacteriodota). Details of the marker genes, primer sequences and qPCR reactions are listed in the Supplementary Table S2.

Quantitative PCR assays were conducted using the SybrGreen Mix kit (Invitrogen) on BioRad CX96 instrument under the following conditions: an initial step at 95°C for 10 min, followed by 40 cycles of 95°C for 15 s and 60°C for 1 min, followed by the completion of a melting curve from 55°C to 95°C in 0.5°C increments. Reactions were carried out in 25 μL volume, with forward and reverse primers at a final concentration of 200 nM (Eurogentec, France), and 2 μL of DNA template. To prevent inhibitors from affecting the PCRs, DNA samples were diluted 10- and 100-fold and the weaker dilution without inhibitors was retained.

Negative controls (without template DNA) were included in triplicate for each run. A gBlocks Gene fragment (IDT, Integrated DNA Technology) containing a 406 bp partial sequence of 16S rRNA gene belonging to the Bifidobacteriaceae family was used as standard. This synthetic oligonucleotide was used at 10-fold dilutions ranging from 10^6^ to 1 copies per qPCR. PCR efficiency varied between 85.1 and 99.8% and coefficient of determination (*R*^2^) for all the standard curves were > 0.99.

Then, the sensitivity and specificity of the primers targeting the ASV belonging to the family Bifidobacteriaceae were assayed on target (*n* = 83) and non-target fecal (*n* = 54) samples using qPCR assays.

## 3 Results

### 3.1 Bacterial communities in seal feces

#### 3.1.1 Analysis of raw data of metabarcoding 16S

Illumina sequencing of the V3-V4 hypervariable region of the 16S rRNA gene yielded a total of 5,820,125 reads for the 125 seal fecal samples. After verification of sequence quality, assembly and chimeras, a range of 3,340 to 80,009 reads per sample with an average of 26,325 ± 15,061 reads per sample were retained (56.5% of the raw data) for further analysis. After the dbOTU3, ASV clustering and microDecon steps, a total of 1,184 ASVs were obtained ranging from 21 to 187 per sample, where 98.8% of the ASVs were assigned taxonomically to the phylum level, 91.6% to the genus level, and 61.4% to the species level.

#### 3.1.2 Taxonomic composition of seal fecal bacterial communities

**Figure 2:**
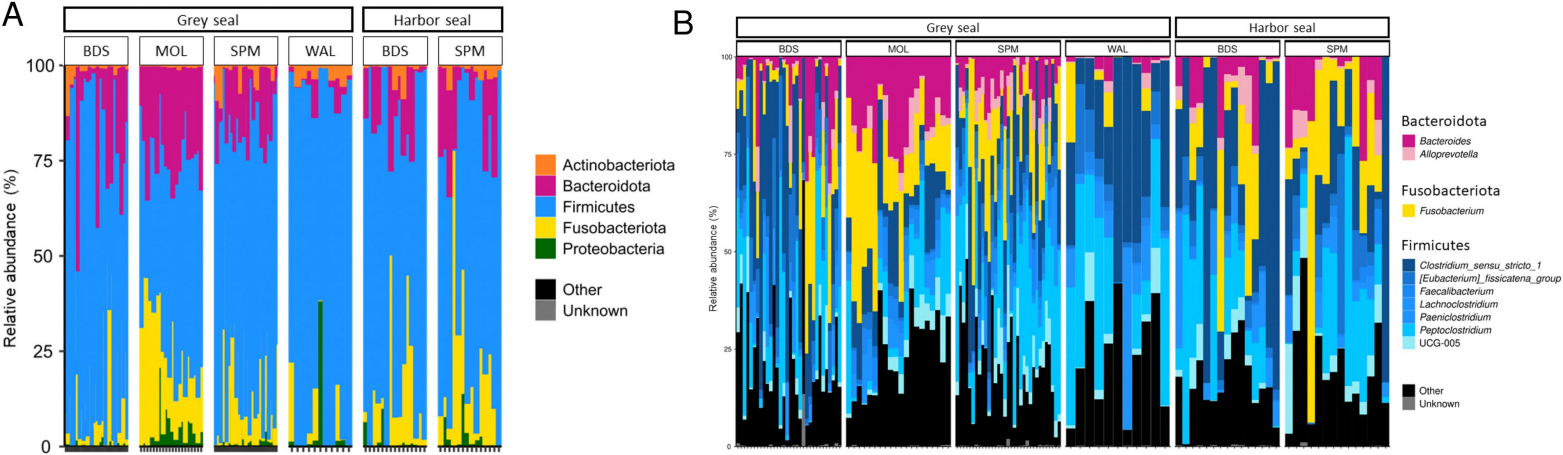
Phylum-level (A) and genus-level (B) bacterial community composition in two seal species: grey seal (*n* = 96) and harbor seal (*n* = 29). BDS (Baie de Somme); WAL (Walde), MOL (Molène archipelargo), Saint-Pierre et Miquelon (SPM, Le grand barachois, Miquelon).

Five phyla were found to be the most abundant in the feces of grey seals (*n* = 96): Firmicutes (67.3% ± 4.4%), Fusobacteriota (15.4% ± 24.6%), Bacteroidota (13.1% ± 19.8%), Proteobacteria (2.7% ± 7.9%) and Actinobacteriota (1.5% ± 3.4%; Figure 2A).

These five phyla were also the most abundant in the feces of harbor seals (*n* = 29) with similar values: Firmicutes (74.1% ± 65.9%), Fusobacteriota (12.9% ± 15%), Bacteroidota (10.2% ± 14.2%), Proteobacteria (1.6% ± 2.7%) and Actinobacteriota (1.2 ± 2.1; Figure 2A).

In the grey seal, the main genus found was the genus *Clostridium sensu stricto* 1 (17.8% ± 11.1%), followed by the genus *Peptoclostridium* (16.4% ± 11.3%), *Fusobacterium* (14.8% ± 13.0%) and, to a lesser extent, *Bacteroides* (9.1% ± 8.8%), [Eubacterium] Fissicatena group (5.6% ± 3.9%), *Ruminococcaceae* UCG-005 (4.1% ± 3.8%), *Paeniclostridium* (2.6 ± 3.0%), *Faecalibacterium* (3.3% ± 2.8%) and *Alloprevotella* (2.2 ± 2.2%; Figure 2B).

In the harbor seal, a similar distribution of genera was observed with the main genus found *Clostridium sensu stricto* 1 (28.7% ± 29.1%), followed by the genera *Peptoclostridium* (14.8% ± 11.5%), *Fusobacterium* (12.1% ± 8.1%) and, to a lesser extent, the genera *Bacteroides* (6.9% ± 6.31%), [Eubacterium] Fissicatena group (5.4% ± 5.5%), *Ruminococcaceae* UCG-005 (3.7% ± 2.9%), *Paeniclostridium* (3.7% ± 5.9%), *Alloprevotella* (2.3% ± 2.2%) and *Faecalibacterium* (2.9% ± 2.2%; Figure 2B).

**Figure 3:**
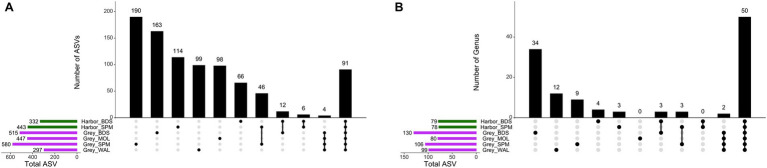
UpSetR plots showing the number of unique (single dot), shared (connected dots), and total (horizontal bars) ASVs (A) and genera (B) with an 80% prevalence in the fecal samples.

Of the genera identified, 34.4% occurred exclusively in one seal species and one site, while 27.8% occurred in both two species and different sites (Figure 3B). For ASVs, the majority of ASVs are found exclusively in one species and one site (57.9%), while only 91 ASVs (7.2%) are shared between the two species and the four sites (Figure 3A).

The core group of harbor seals consisted of nine ASVs belonging to the [Eubacterium] fissicatena group, *Blautia, Clostridium sensu stricto* 1 (two ASVs), *Fusobacterium* (two ASVs), *Peptoclostridium*, *Ruminiclostridium* 9, *Ruminococcaceae* UCG-005. The core group of grey seals consisted of these nine ASVs and a further five ASVs belonging to the genera *Bacteroides, Faecalibacterium, Fournierella, Lachnoclostridium* genera and to the family Erysipelotrichaceae.

#### 3.1.3 Alpha diversity

**Figure 4:**
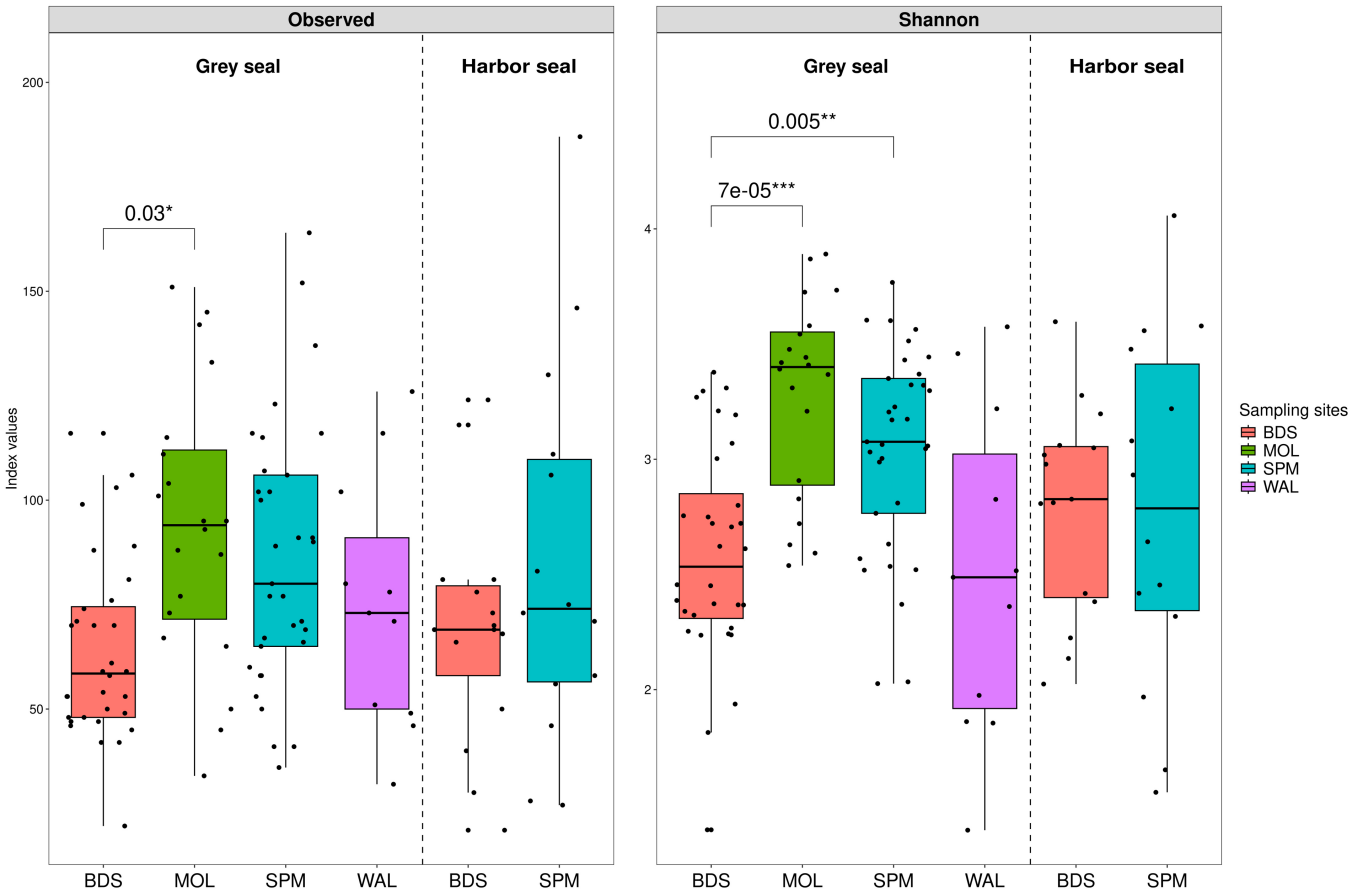
Boxplots illustrating alpha diversity in grey seal (n = 96) and harbor seal (*n* = 29) feces. The following indices were calculated: the observed species richness (A) and the Shannon diversity (B) index calculated from a contingency table of ASVs.

Two indices were calculated to evaluate alpha diversity within the analyzed dataset: the observed species richness (quantitative species richness) and the Shannon index (non-parametric quantitative species richness) (Figure 4).

The observed species richness index shows a similar alpha diversity for grey seals, ranging from 22 to 164 (median value 73), and harbor seals, ranging from 21 to 187 (median value 71) (Figure 4A). The Adonis statistical test concludes that there is no significant difference between the two seal species (*p*-value 0.54074 > 0.05).

However, a slight significant difference was observed according to the sampling location: BDS, SPM, WAL, and MOL (Adonis p-value 0.01768). The pairwise t-test shows a difference between grey seals (GS) from MOL and grey seals from BDS (*p*-value 0.03) [GS-MOL from 34 to 151 (median 94) versus GS-BDS from 22 to 116 (median 58.5)].

Similarly, for the Shannon index, the Adonis statistical test shows that there is non-significant difference between the two seal species (*p*-value 0.37156; Figure 4B). The Shannon index varies for grey seals from 1.4 to 3.9 (median 2.9) and for harbor seals from 1.5 to 4.1 (median 2.8). However, a significant difference was observed according to the sampling location: BDS, SPM, WAL, and MOL (Adonis *p*-value 0.00697). The pairwise t-test shows a difference between grey seals from MOL and from BDS (*p*-value <0.001) [GS-MOL from 2.5 to 3.9 (median 3.4) versus GS-BDS from 1.4 to 3.4 (median 2.5)], and between grey seals from BDS and SPM (*p*-value 0.005) [GS-BDS from 1.4 to 3.4 (median 2.5) versus GS-SPM from 2.0 to 3.8 (median 3.1)].

#### 3.1.4 Beta diversity

**Figure 5:**
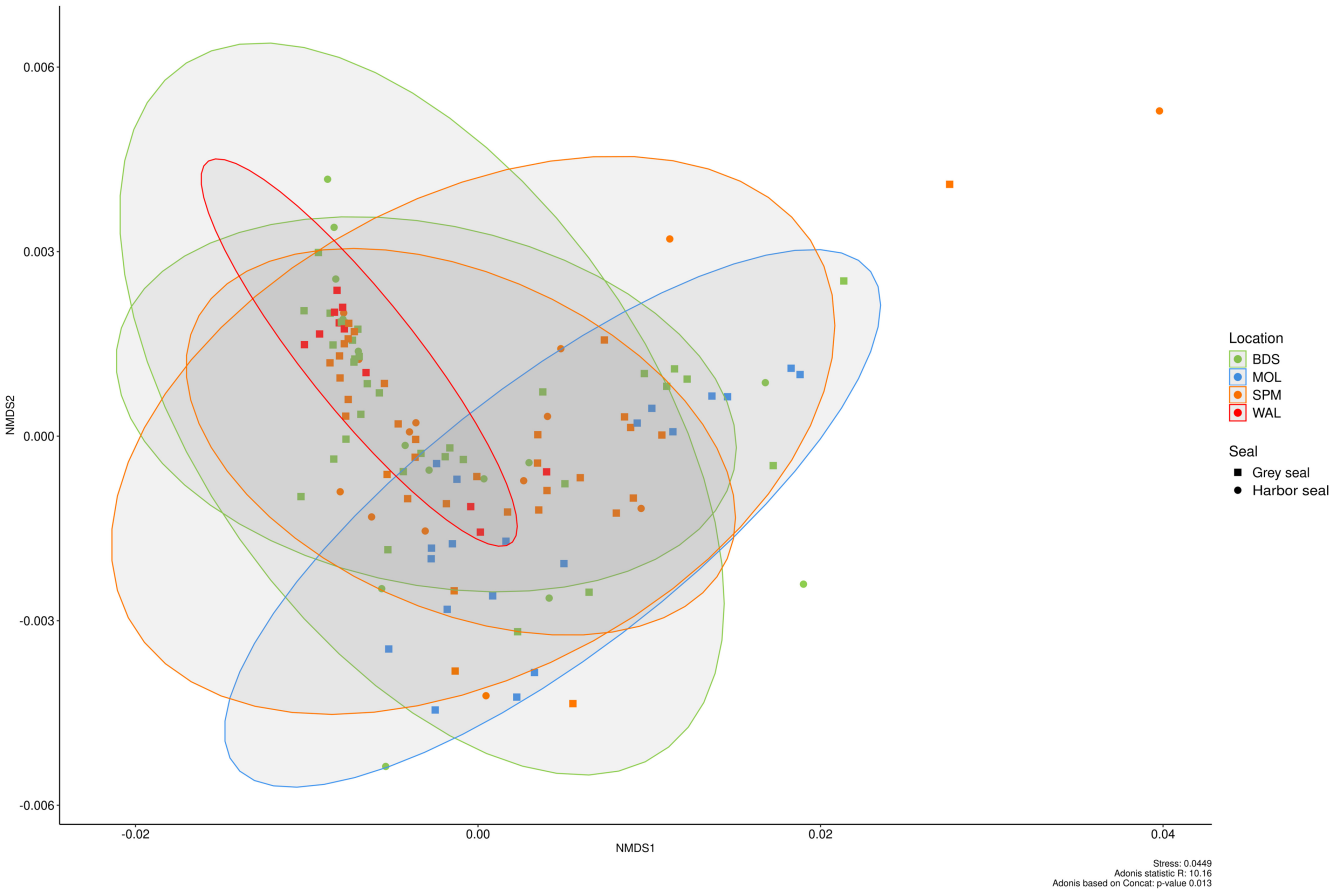
Beta diversity in the feces of two seal species (grey seal and harbor seal) collected in four sites (BDS, POL, WAL, and SPM) represented as NMDS obtained from a distance matrix calculated with the weighted UniFrac index. The data were previously normalized with CSS.

NMDS (weighted UniFrac index, on CSS normalized data) of the seal feces dataset was used to plot the beta diversity between samples (Figure 5). There was a slight significant difference in the bacterial community between groups (seal species and sites) (Adonis p-value: 0.015). Bacterial communities between the grey seals of MOL were significantly different from those of WAL (pairwise Adonis p.adjusted 0.015).

In fact, higher abundances of Fusobacteriota (23.1% ± 28.5% vs. 4.7% ± 16.9%) and Bacteroidota (22.7% ± 20.3% vs. 2.6% ± 7.8%) were obtained in MOL seal feces whereas a higher abundance of Firmicutes (83.8% ± 31.6% vs. 50.1% ± 46.9%) was observed in WAL fecal samples. More precisely, higher abundances of the genera *Fusobacterium* (22.0% ± 19.4% vs. 3.5% ± 5.3%) and *Bacteroides* (16.8% ± 12.9% vs. 1.6% ± 1.6%) were obtained in MOL seal feces whereas higher abundances of the genera *Clostridium sensu stricto* 1 (28.7% ± 19.7% vs. 11.6% ± 8.5%), *Peptoclostridium* (23.6% ± 13.7% vs. 9.0% ± 11.9%), [Eubacterium] fissicatena group (5.8% ± 4.6% vs. 2.5% ± 2.8%) were obtained in WAL seal feces.

### 3.2 Analysis of viral communities

The two sequencing runs provided about the same number of reads (about 3.8 million of reads for run 1 and 3.1 for run 2). After cleaning and deduplication, reads obtained for each sample triplicates were merged, decreasing the variability of read numbers obtained for each library. After this step, number of reads varied from four to 10 million per sample. More than 50% of the samples (24 samples out of 42 samples) displayed number of reads comprised between five to 7 million. This is an important point as despite efforts during sample preparation, the read number identified as bacteria was still high and represented 64% of total read numbers. Only three samples provided less than 10 thousand viral reads, while 24 samples displayed more than 100 thousand reads (data not shown). Overall, viral reads represent around 6%, and were identified as belonging to different viral families, and except two samples (Fph102 and Fph106) all samples provided enough reads to build contigs and identified some viral families.

**Figure 6:**
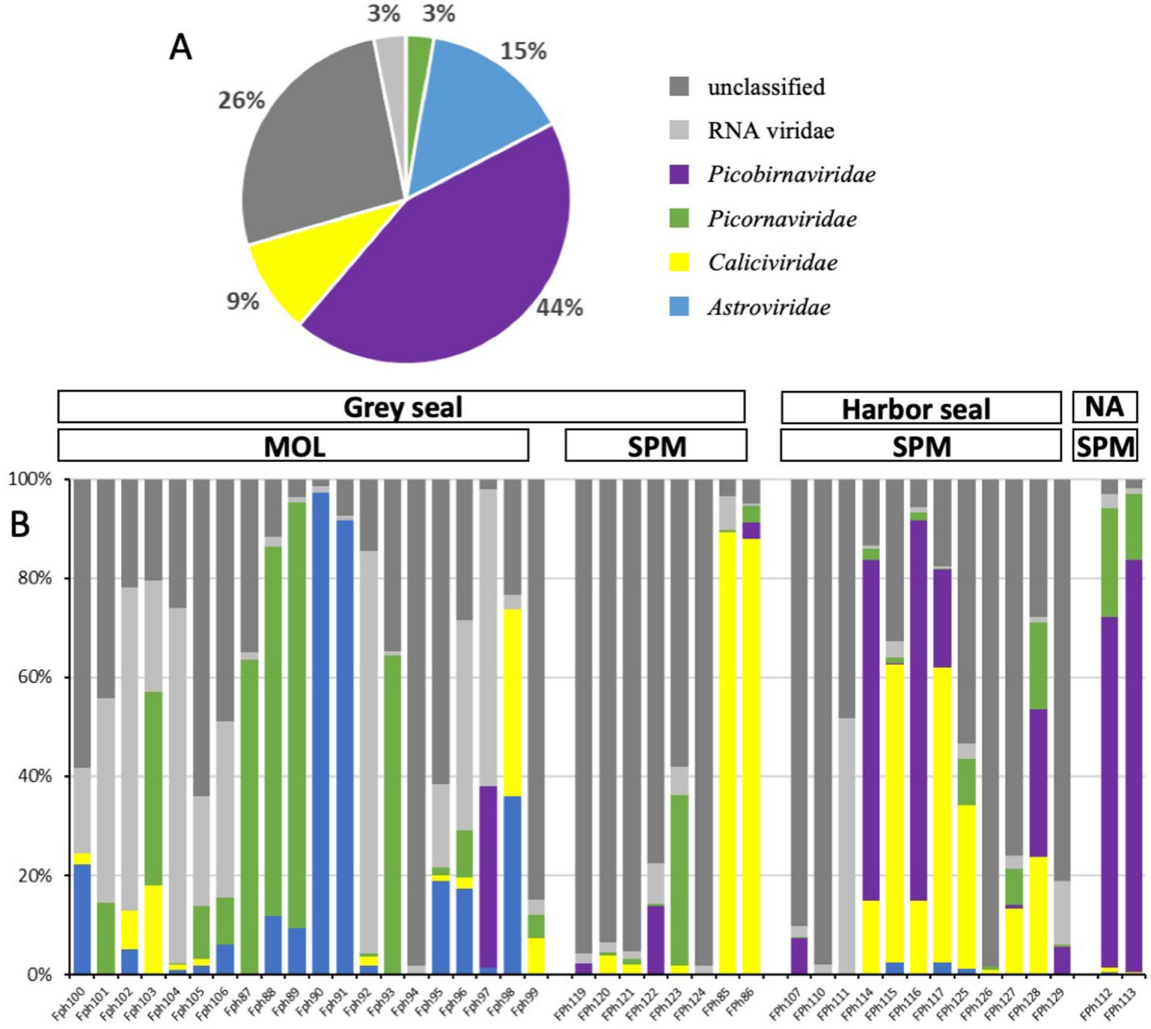
Viral reads distribution among viral families for the two seal species and the two sample sites. (A) Distribution of cumulative reads obtained for all samples expressed in % for the different families. (B) Relative abundance of the different families identified in each sample including unclassified or RNA *viridae* reads.

**Figure 7:**
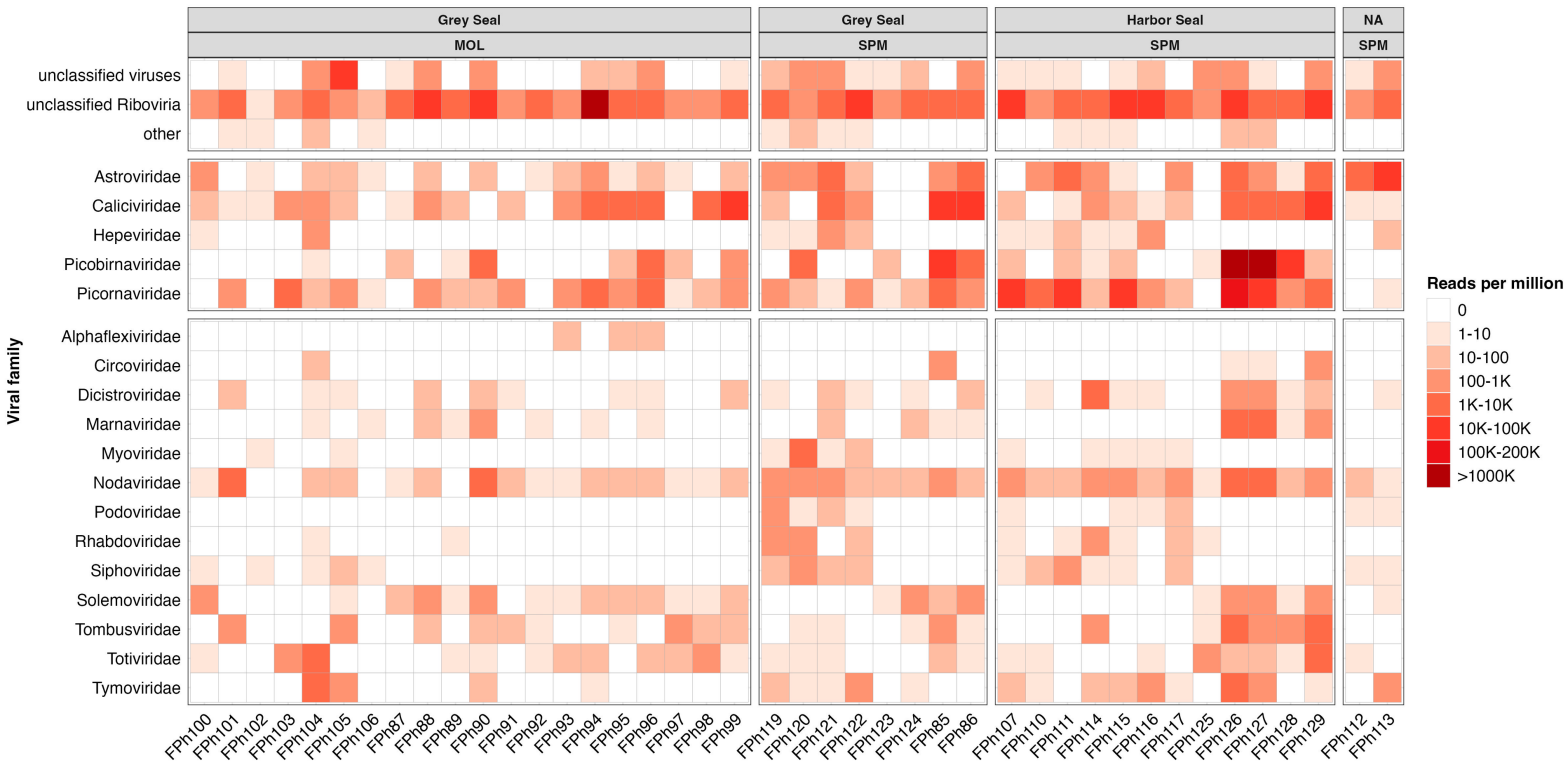
Heatmap representing the abundance of the 13 most abundant families and the other viral read abundance all included as ‘other families’, the scale bar expressed the number of reads par million (rpm).

**Figure 8:**
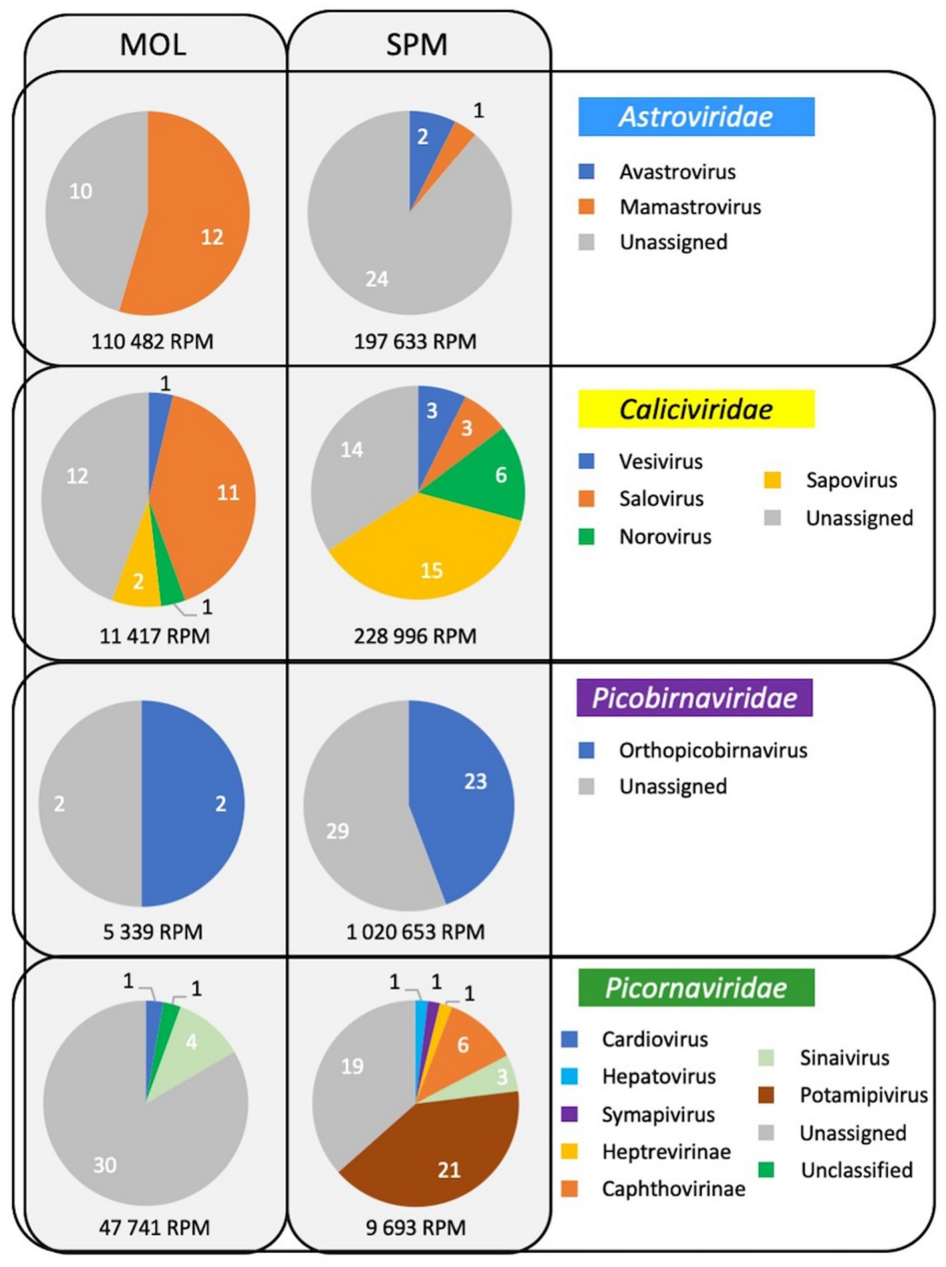
Viral diversity identified in samples collected in the two sites for four families infecting mammals.

As frequently observed with virome analysis, many reads (26%) cannot be classified, raising the need to identify all these data (Figure 6A). Reads belonging to diverse families such as *Discistroviridae*, *Nodaviridae*, *Marnaviridae*, *Totiviridae*, *Tymorviridae*, *Tombusviridae*, *Solemoviridae*, represented 23% of total viral reads, being the only reads identified in some samples (Figure 6B; Figure 7). Within these families, some were identified in all samples as for the *Nodaviridae* or *Picornaviridae* families or, to a lesser extent, the *Tymoviridae* family. Many reads (44%) were identified as belonging to the *Picobinaviridae* family (Figure 6A). This percentage varied among the different samples but seems to be more frequently detected in samples collected in SPM. This viral family that includes small viruses with a segmented dsRNA genome, was first suggested to infect mammals but now has been described in a large number of invertebrates and bacteria. Recently, an emerging picobirnavirus genotype was identified in patient with an acute respiratory virus, with presumably zoonotic origin (Berg et al., 2021). Then most of the reads were identified as belonging to the *Astroviridae* reads (15%) or *Caliciviridae* reads (9%) (Figure 6A). *Picornaviridae* reads were identified mainly in grey seal from MOL, but most of contigs could not be further identified, while in samples collected in SPM site, some contigs belonging to different genera were identified (Figure 8). *Caliviridae* reads were predominantly identified in samples collected in SPM whatever the seal species but the observed diversity was comparable in the two sites with contigs belonging to different genera identified (Figure 8). Reads identified as belonging to the *Astroviridae* family were detected in a large range of samples (Figure 7). Within this family, most of the reads were unassigned, however mamastrovirus reads were identified mainly from MOL samples. This family represented 98% of the reads detected in one sample (Fph90), and almost 92% of the reads of another sample (Fph91) (Figure 6B).

### 3.3 Animal microbial communities and MST marker

#### 3.3.1 Comparison of bacterial communities of seal fecal samples to other animal fecal sources in metropolitan France

In order to identify the specificities of seal fecal microbial communities and develop thus a seal-associated MST marker, the bacterial communities of seals were first compared to those of wild waterbirds and livestock (i.e., cattle and pigs).

**Figure 9:**
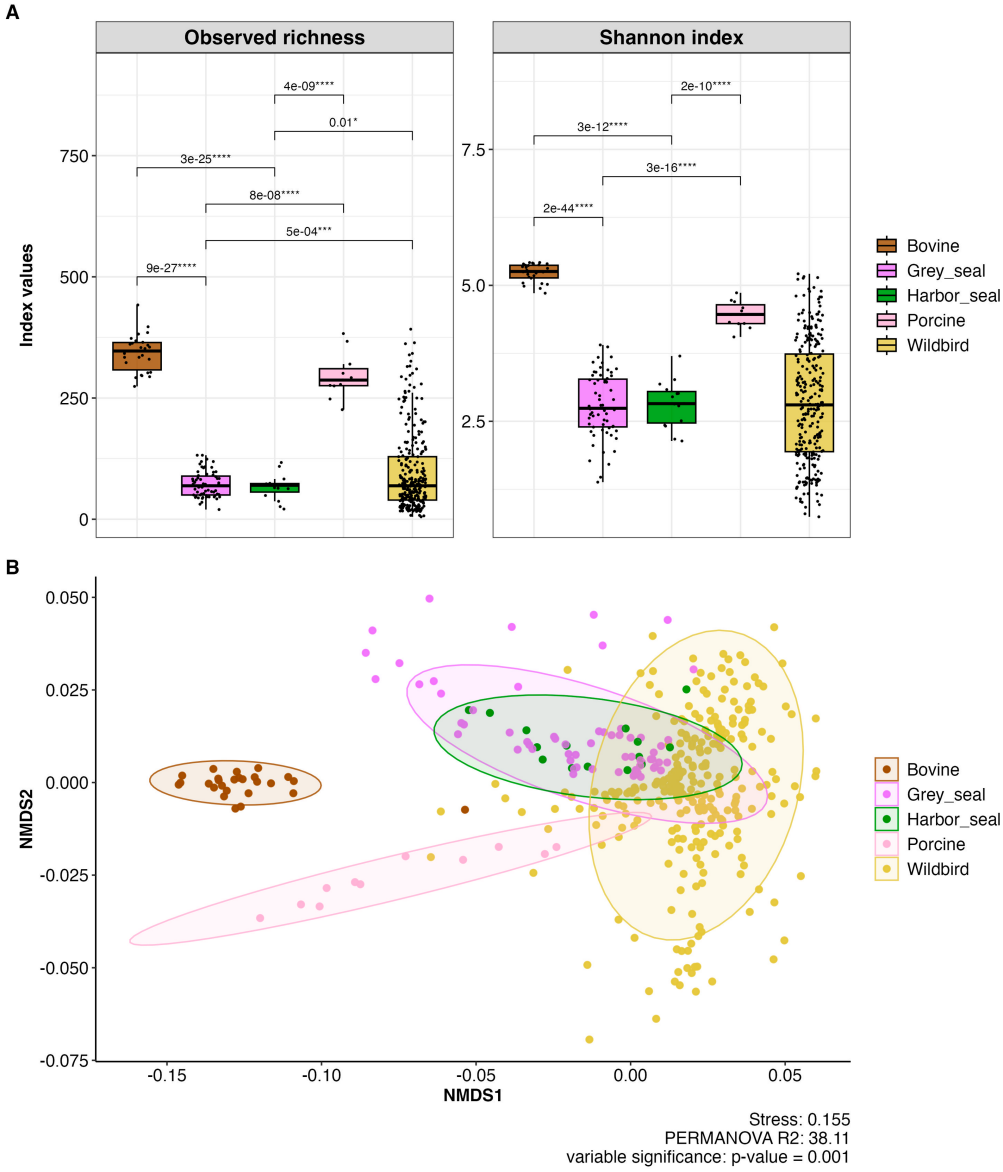
(A) Boxplots showing alpha diversity (observed species richness and Shannon indices) in fecal samples of grey and harbor seal fecal samples (*n* = 78), wild waterbirds (wildbird; *n* = 275), cattle (bovine; *n* = 26), pigs (porcine; *n* = 11) (total number of samples n = 390; metropolitan France). (B) Beta diversity in the feces of seals, wild waterbirds, cattle and pigs represented as NMDS obtained from a distance matrix calculated with the weighted UniFrac index. The data were previously normalized with CSS.

The observed species richness and Shannon indexes show low alpha diversity in the grey and harbor seal feces similar to those observed previously for wild waterbird fecal samples and significantly different from the high ones observed in the cattle and pig samples (Adonis statistical test; *p* < 0.0001; Figure 9A). For example, the Shannon index of the cattle fecal samples ranged from 4.8 to 5.4 (median value 5.3) whereas in the grey seal feces samples the Shannon index ranged from 1.4 to 3.9 (median value 2.8).

Concerning beta diversity, NMDS analysis of the entire dataset using the weighted UniFrac distance metric demonstrated that individuals from the same group (i.e., grey seals, harbor seals, wild waterbirds, cattle, or pigs) clustered together. However, there was notably greater diversity among the wild waterbird samples (stress value 0.157; Permanova; *R*^2^ = 52.21; *p* = 0.001; Figure 9B).

The bacterial communities observed in grey seal and harbor seal feces are significantly different from those present in the fecal samples of all the other animals (i.e., cattle, pig, and wild waterbirds) (pairwise- Adonis *p*-value: 0.001 and p.adjusted 0.01).

The ANCOM (Analysis of Composition of Microbiomes) analysis conducted on the five groups (i.e., grey seals, harbor seals, wild waterbirds, pigs, and cattle) revealed that the seal groups are distinguished from the others primarily by a significantly higher presence of sequences from the following genera: the [Clostridium] inoculum group, [Eubacterium] Fissicatena group, *Lachnoclostridium, Peptoclostridium*, *Tuzzerella*, belonging to Firmicutes; *Anaerobiospirillum* and *Edwardsiella*, belonging to Proteobacteria; *Fusobacterium,* belonging to Fusobacteroidota; and *Atopobium*, belonging to Actinobacteriodota (Supplementary Figures S1A,B).

Furthermore, we can note that the fecal samples closer to seal samples belong to wild waterbirds and more precisely shorebirds such as knots, oystercatchers, and curlews (Supplementary Table S1).

In addition, ANCOM identified 83 ASVs that were present only in seal feces and/or at higher levels in seal feces than in other animal sources. The 20 most specific ASVs for seals were selected and pairs of primers were designed targeting seal-associated members of the genera *Fournierella, Atopobium, Slackia* and the family Bifidobacteriaceae (i.e., four of these ASVs) (Supplementary Table S2).

#### 3.3.2 Seal-associated bacterial MST qPCR marker selected

The seal-associated marker targeting the Bifidobacteriaceae family (Seal_Bifido) was found to be the most efficient. This MST marker presented a sensitivity of 89.8% on grey seal feces (*n* = 49) [i.e. 82.6% on seal feces from BDS (*n* = 23); 95% on seal feces from MOL (*n* = 20), and 100% on seal feces from SPM (*n* = 6)] while it presented a sensitivity of 100% on harbor seal feces (*n* = 14) from SPM and no harbor seal feces (*n* = 15) from BDS was found positive for this MST marker (only one detected). A specificity of 97.1% was obtained when tested on 69 non-target fecal samples [human feces (*n* = 9), WWTP effluent (*n* = 4), cattle fecal samples (*n* = 9), pig fecal samples (*n* = 10), horse feces (*n* = 9), poultry (*n* = 4; chicken and guinea fowl), pigeon (*n* = 1), wild waterbirds (*n* = 23; mallard, *n* = 3; knot and dunlin, *n* = 3; Brent goose, *n* = 3; seagull, *n* = 3; oystercatcher, *n* = 5; common shelduck, *n* = 3; great cormorant, *n* = 3)]. Only two cormorant feces (*n* = 3; FO199 and FO211) were found positive with the seal-associated marker. Interestingly, the melt curve for these non-target samples showed a single peak at a slightly different melting temperature (Tm) to that obtained for the seal feces samples (i.e., 85.5°C vs. 84.5°C).

#### 3.3.3 Viral MST marker

Unlike the bacterial communities, the analysis of the RNA virus communities obtained in this study did not allow us to identify viral sequences specifically associated with seals that could be used to develop a viral qRT-PCR MST marker. This is due to the fact that viral communities were not characterized in other animals in this study and the percentage of unassigned reads obtained was high.

## 4 Discussion

This study describes the microbial communities (both bacterial and viral) present in the two most common seal species in France (i.e., grey seal and harbor seal) and across several contrasting sites. It included 132 fecal samples (from 96 grey seals, 29 harbor seals, and seven unspecified seal species) collected from various locations, including SPM and three French mainland sites. The inclusion of a large number of fecal samples from wild free-ranging seals and the analysis of both bacteria diversity and virus diversity set this study apart from previous research, which often had a smaller sample size, and frequently from dead or sick individuals. In our study, samples were collected randomly without any *a priori* of the status health of the seals.

### 4.1 Bacterial communities in seals compared to other sources

The fecal bacterial communities found in seals were significantly different from those of terrestrial domesticated mammals such as pigs and cattle (omnivorous and herbivores, respectively) and wild waterbirds in France (Boukerb et al., 2021). This difference in bacterial communities between marine and terrestrial mammals was also previously described and can be attributed to a significantly higher relative abundance of members of the Fusobacteroidota phylum in seals compared to terrestrial mammals (Nelson et al., 2013; this study). Such results were observed in previous studies on harbor seals (Switzer et al., 2023), Australian fur seals (Glad et al., 2010; Nelson et al., 2013; Smith et al., 2013) and sea lions (Bik et al., 2016).

Members of the Fusobacteroidota phylum are gram-negative bacteria that range from facultative anaerobes to obligate anaerobes. They ferment carbohydrates or amino acids to produce various organic acids, including acetic, formic, and butyric acid (Bennett and Eley, 1993; Olsen, 2014). These bacteria can be found in various habitats, such as sediments and the gut microbiomes of strict carnivores adapted to diets rich in proteins, purines, and polyunsaturated fatty acids.

Furthermore, this study enabled the comparison of bacterial communities of wild marine mammals and wild waterbirds from coastal areas, which had not been done to the best of our knowledge. The bacterial communities in seals were found to be more similar to those of wild waterbirds, especially shorebirds (as shown in the NMDS Figure 9), than to those of terrestrial mammals (i.e., pigs and cattle). Both seals and wild waterbirds exhibited a high average relative abundance of members from the phyla Firmicutes and Fusobacteriota, as well as the following genera: *Clostridium sensu stricto* 1, *Peptoclostridium, Bacteroides, Fusobacterium,* and *Cetobacterium*. However, wild waterbirds had a greater relative abundance of members from the phyla Proteobacteria and Actinobacteriota compared to seals.

### 4.2 Bacterial communities in seals

This study described the bacterial communities of grey seals and harbor seals. To our knowledge, only two studies reported bacterial data on grey seals (based a single seal, not using metabarcoding 16S, and focused on seal pups and yearlings, respectively) (Glad et al., 2010; Watkins et al., 2022), whereas the gut microbiota of harbor seals has been described in several studies (Numberger et al., 2016; Pacheco-Sandoval et al., 2019; Pacheco-Sandoval et al., 2022; Switzer et al., 2023).

Five main phyla (i.e., Firmicutes, Fusobacteriodota, Bacteroidota; Proteobacteria, and Actinobacteria) were observed in both grey seals and harbor seals in this study, consistent with previous studies on different seals species (Glad et al., 2010; Tian et al., 2022). However, the relative abundance of these main phyla differed in the feces of harbor seals in France compared to those of harbor captive seals in Germany (Baltic sea) or harbor wild seals in Mexico (Baja California) (Numberger et al., 2016; Pacheco-Sandoval et al., 2019). Specifically, the abundance of Firmicutes was nearly twice as high in the feces of French harbor seals (SPM; 65.4% ± 23.3%; *n* = 14 and BDS; 74.1% ± 24.0%; *n* = 15) compared to German (32.2%; *n* = 5) and Mexican (21–57%; five different seal colonies; *n* = 20) harbor seals. On the other hand, the relative abundance of the phylum Bacteroidota (SPM: 12.3% ± 11.6% and BDS: 9.8% ± 9.6%), was approximately half that of German and Mexico seals (27.7% and 19–36%, respectively). The relative abundance of Fusobacteriota (SPM: 18.2% ± 20.0% and BDS: 13.2% ± 15.7%) was lower than the one of German seals (27.3%) whereas the relative abundance of Fusobacteriota in Mexico seals was variable according to the seal colonies (4–42%).

Furthermore, the high Firmicutes to Bacteroidota ratio observed both in grey seals and harbor seals has been previously reported in grey, harbor, and spotted seals (Glad et al., 2010; Numberger et al., 2016; Pacheco-Sandoval et al., 2019; Tian et al., 2022). This high ratio observed may be related to their fat reserves (blubber).

There was a significant inter-individual variability in bacterial communities among the seal feces collected in this study, consistent with previous studies (Pacheco-Sandoval et al., 2019). The difference in relative abundance can be partly explained by the diet, which is known to be one of the main factors impacting the composition of the gut microbiota. In fact, diet of seals varies geographically, seasonally, inter-annually, but also inter-individually (Brown and Pierce, 1998; Gosch et al., 2019; Walton and Pomeroy, 2003; Wilson and Hammond, 2019). Seals are opportunistic hunters and adapt their carnivorous diet to local conditions, specializing in different hunting techniques, such as shredding fishing nets or traps containing shellfish. In addition to diet, other factors can contribute to the inter-individual differences in bacterial communities. These include variations in seal populations and species, the behavior and habitat of seals, such as wild free-ranging behavior and the type of habitat [e.g., sand (SPM, BDS and WAL) or rocks (MOL)]. Furthermore, individual characteristics such as age, sex, and health status can impact microbial composition. For example, Tian et al. (2020) and Pacheco-Sandoval et al. (2022) identified age-related differences in the composition of the gut microbiota in spotted seals (*Phoca largha*) and harbor seals in Mexico, respectively and Switzer et al. (2023) sex- and age-related differences in the composition of rectal swabs in harbor seals (*Phoca vitulina richardii*) in California.

Unfortunately, despite collecting freshly collected feces from the environment throughout the study, host characteristics such as age, behavior and health status could not be obtained. Among the available parameters, a significant difference in bacterial communities was observed based on site (i.e., grey seals from WAL and MOL), but no significant differences were found between the two seal species.

The fecal microbial communities can also be used to study the presence of bacteria responsible for zoonoses in marine mammals and human pathogenic bacteria in these seals, which could be sentinels for marine and human health in coastal areas.

Genera already isolated from seals such as *Bisgaardia* spp., *Campylobacter* spp., *Clostridium* sensus stricto 1 including *Clostridium perfringens*, *Edwardsiella* spp., *Mycobacterium* spp.*, Mycoplasma* spp.*, Photobacterium damnselae*, streptococci, and *Vibrio* spp. as well as genera of the family Erisipelotrichaceae, all known to contain marine mammal or human pathogenic species were found in the present study (Waltzek et al., 2012; Hughes et al., 2013; Greig et al., 2014; Lisle et al., 2004; Nelson et al., 2013; Stoddard et al., 2007; Siebert et al., 2017; Pacheco-Sandoval et al., 2024).

However, other genera also responsible for zoonotic diseases such as *Brucella* spp., *Klebsiella pneumoniae*, *Leptospira* spp., *Pleisomonas shigelloides*, *Salmonella* spp., *Staphylococcus aureus* (Waltzek et al., 2012; Greig et al., 2014; Siebert et al., 2017) were not found in these seal bacterial communities. One explanation could be that these feces were from healthy animals and the bacteria belonging to these genera were absent or present at very low concentrations in the feces analyzed in this study.

Fecal indicator bacteria (FIB) such as *Escherichia coli* (*E. coli*) are present in seal feces, as they are in the feces of other marine and terrestrial mammals (Fulham et al., 2020). Concentrations of *E. coli* ranging from 2.5 10^6^ ± 4.9 10^6^ CFU/g of feces were found in the feces of SPM seals (*n* = 28) in 2020 (data not shown), consistent with those ranging from 4.0 10^6^ to 9.2 10^8^ MPN per g of feces (*n* = 10) found in harbor seals in the United States (Calambokidis and McLaughlin, 1987). In addition, the genus *Escherichia/Shigella* was reported in the seal bacterial communities in this study, as previously reported in seals in Germany, Antarctica, Scotland, and the United States (Nelson et al., 2013; Greig et al., 2014; Numberger et al., 2016; Switzer et al., 2023).

The ubiquitous presence of *E. coli* in seals, as well as in other fecal sources highlights the need to develop host-associated MST bacterial markers to identify potential sources of *E. coli* contamination in coastal bathing areas and shellfish-harvesting areas.

### 4.3 Viral communities in seals

As mentioned above, there is a paucity of data on the seal virome in the literature. Despite methodological improvements in recent years, virome identification is still challenging. Indeed, most viruses are small particles with short genomes and the amount of unknown or unclassified sequences is very important, as highlighted by the *Tara* Oceans expeditions (Alberti et al., 2017). One possible approach is to enrich for viral sequences during the library preparation using probes targeting a large diversity of viral sequences (Briese et al., 2015). Such an approach was found to be valuable in our hands to identify sequences related to human viruses in bivalve mollusks exposed to wildlife (Bonny et al., 2021), and was used in seal samples (serum and feces) collected in an area with a high human density (Martínez-Puchol et al., 2022). As our study aimed to assess the diversity of viral sequences in an area with a very low human population density such as Saint-Pierre et Miquelon, we did not perform any enrichment step during the library preparation. This may explain why a large number of reads were identified as fish viruses or phages. Such a preponderance of phages or unknown riboviruses was previously described in subantartic and South American fur seals, where up to 89% of reads were identified as phages (Kluge et al., 2016). Our results are more comparable to their findings in *Arctocephalus tropicalis* than to the virus distribution obtained on samples collected from *Arctocephalus australis* (Kluge et al., 2016). Nevertheless, our data showed a high percentage of reads identified as belonging to four viral families, that we selected because they can infect mammals but also humans, such as *Picobirnaviridae*, *Picornaviridae*, *Caliciviridae*, and *Astroviridae*. These four families accounted for approximately 3 to 8% of the reads from fecal samples collected from subantarctic and South American fur seals (Kluge et al., 2016). As with bacteria, some differences between the two collection sites can be highlighted, with *Astroviridae* and *Picornaviridae* reads being more frequently identified at one site. However, the relatively small number of samples collected prevents any further analysis of such distributions between sites or seal species. The detection of picornaviruses in marine mammals has been described previously (Kapoor et al., 2008). More recently, some strains have been characterized such as an aquamavirus A, with the description of two novel strains, such as a harbor seal picornavirus and a ribbon seal picornavirus (Rodrigues et al., 2020). In our study, some sequences related to potamipivirus, a genus related to the aquamivirus were detected in the SPM site, but a more precise identification cannot be obtained. Some other RNA viruses such as the phocine distemper virus which belongs to the morbilivirus (*Paramyxoviridae* family), have been responsible for large outbreaks in different seal populations, but as enveloped viruses they may be less resistant in stool samples (VanWormer et al., 2019). As mentioned above, no data were collected on the health status of the seals, so the sequences detected may be associated with healthy seals and very little is known about viruses that cause disease in seals. In this study, we focused on RNA viruses as they are more likely to mutate than DNA viruses and can jump from one species to another. Recent works have shown that seals are infected and die from some avian influenza viruses or the human pandemic H1N1 virus (Liang et al., 2023; Mirolo et al., 2023; Plancarte et al., 2023). If the route of transmission from humans to seals is not clear, the reverse route (seals to humans) can be hypothesized through the consumption of foods such as shellfish, justifying the four viral families selected for this study, for which the enteric route is the main route of transmission (Santiago-Rodriguez and Hollister, 2023). For example, it might be interesting to find some mamastrovirus or norovirus contigs, as these viruses are also frequently detected in humans. In any case, our study therefore confirms the need to further investigate the degree of homology of these viral sequences with other known viruses that may be detected in coastal environments. For example, it has been hypothesized that some of the vesiviruses (*Caliciviridae* family) may have originated from marine reservoirs, with the detection of antibodies against the San Miguel sea lion viruses (a marine calicivirus) raising the possibility of the emergence of new strains (Smith et al., 1998).

### 4.4 Microbial source tracking seal marker

The difference between seals, wild waterbirds and terrestrial mammals allowed for the identification of specific bacteria to serve as MST markers.

Microbial Source Tracking involves identifying and applying markers associated with different hosts to the environment. Previous studies have developed markers for pigs, cattle, humans, and birds, and the seal marker developed in this study complements the existing panel of markers (Mieszkin et al., 2009, 2010). This study developed the first seal bacterial marker, to our knowledge, and it proved to be sensitive and specific to grey seals. However, the marker was found to be less prevalent in the samples of harbor seals at the BDS site.

In the future, this marker could be applied to areas with high seal numbers, such as in the Wadden Sea (Brasseur, 2017; Brasseur et al., 2015), and could be used in combination with mitochondrial DNA seal markers that can even identify the species of seals (Arnason et al., 1993), providing a complementary approach to identify a source of seal contamination.

This study did not identify any seal-specific viral sequences that could be used to develop a viral MST marker. However, it provided useful results for future comparison with viral communities from other animal sources. By providing a better identification of unclassified riboviruses or other viral families, the virome could also be useful for marker development (Santiago-Rodriguez and Hollister, 2023). More data are needed to complement such an approach.

## 5 Conclusion

The bacterial communities of grey and harbor seals were not significantly different, which is consistent with the partial overlap in the ecological niches (and more specifically their diet, where they co-occur) as already shown in the Eastern Channel (Planque et al., 2021) and presumed in Saint-Pierre et Miquelon (Vincent et al., 2022). They were characterized by a majority abundance of Firmicutes including the genera *Clostridium sensu stricto* 1 and *Peptoclostridium* followed by Fusobacteriota with the genus *Fusobacterium*, and Bacteroiota with the genus *Bacteroides*. However, variations in bacterial communities between sites and between individuals were observed. A similar observation was also done for the virome, raising questions about sources of contamination for the seals (food, water quality…), but also some relationship between bacteria and viruses, other than phages.

In this study, a sensitive and specific MST qPCR bacterial marker belonging to the Bifidobacteriaceae family was developed, which could be used to identify potential fecal contamination of coastal areas by seals.

## Data Availability

The datasets presented in this study can be found in online repositories. The names of the repository/repositories and accession number(s) can be found at: https://www.ebi.ac.uk/ena, ERP149173.

## Footnotes

^1^https://gitlab.ifremer.fr/bioinfo/workflows/samba

^2^https://blast.ncbi.nlm.nih.gov/Blast.cgi; NCBI nt; retrieved in May 2021

^3^v4.1.0; https://primer3.ut.ee/
