# Supplementary figures and images for: Grey and harbor seals in France (mainland and Saint-Pierre et Miquelon): microbial communities and identification of a microbial source tracking seal marker
Source: Front Microbiol. 2024 Dec 4;15:1484094. doi: 10.3389/fmicb.2024.1484094 (PMC11652528; doi:10.3389/fmicb.2024.1484094)

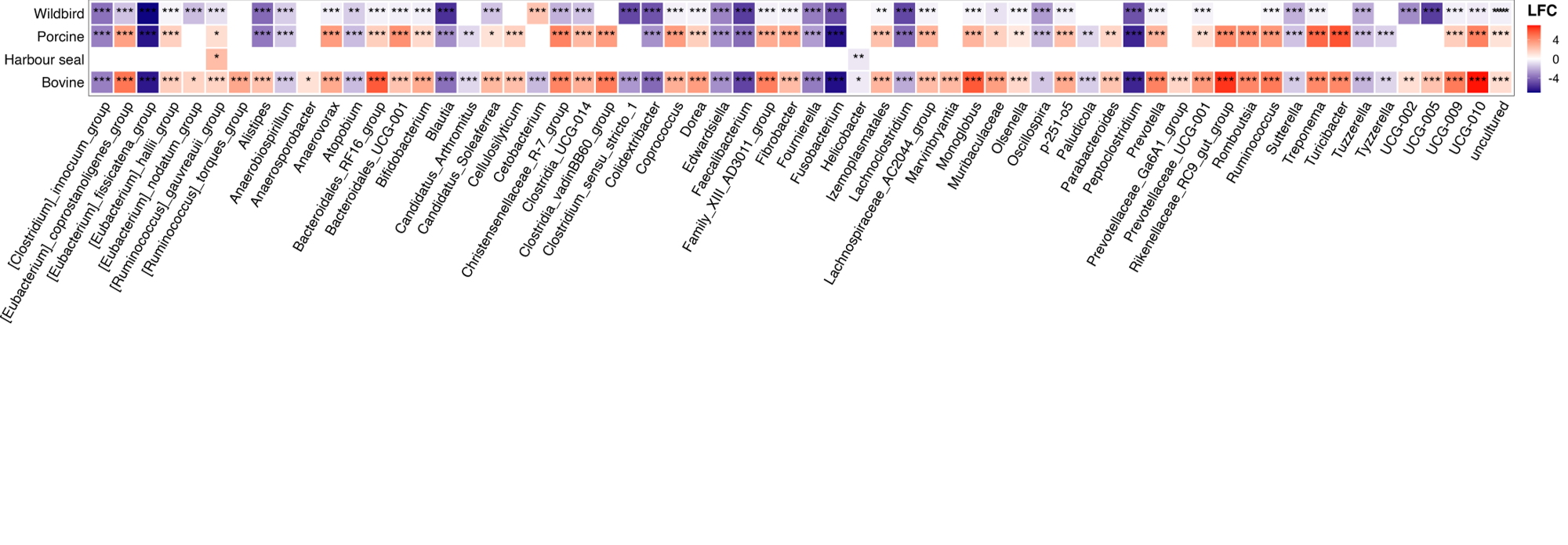

Supplement: Supplementary file 1 [file Image_1.tif]

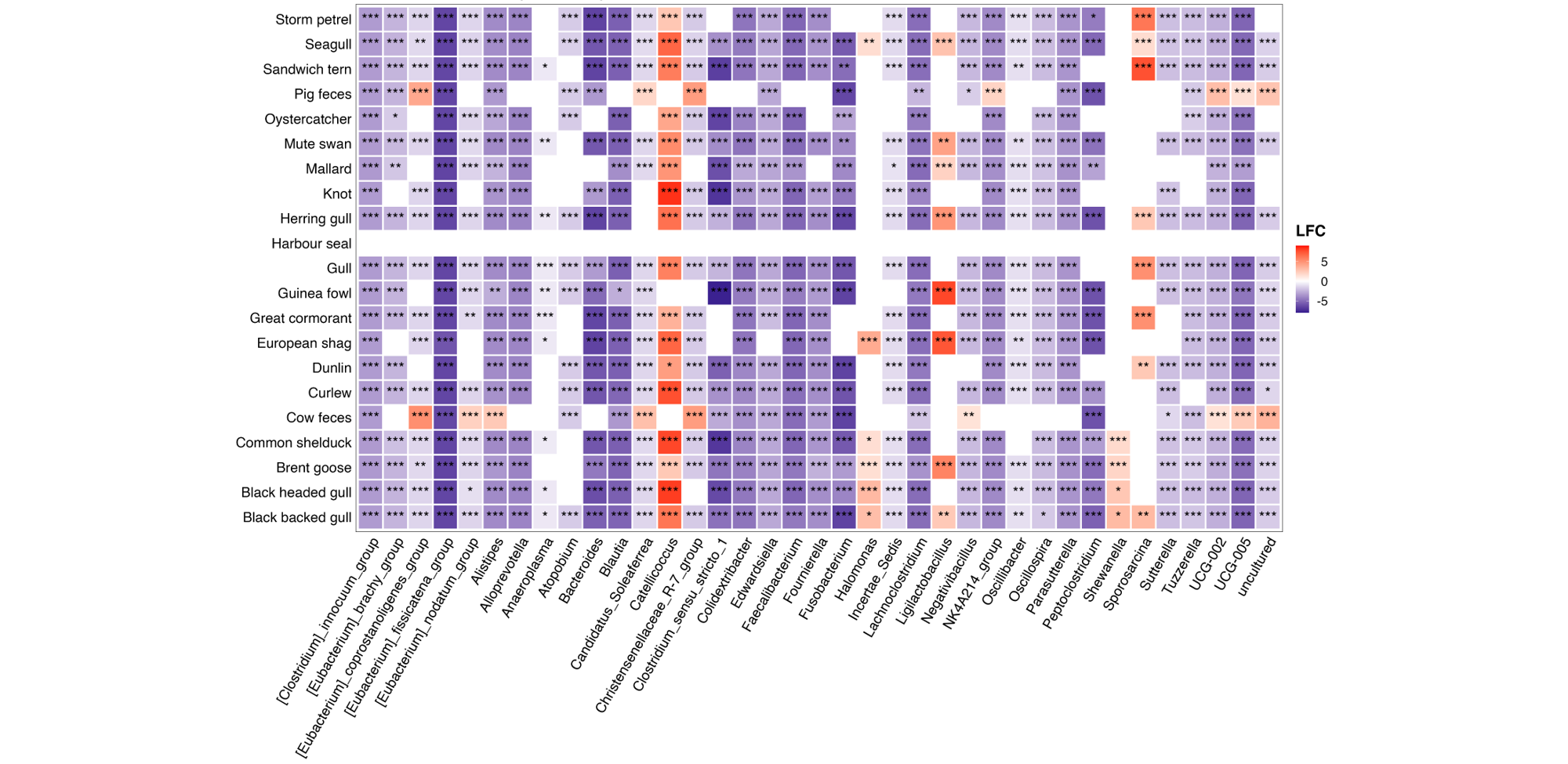

Supplement: Supplementary file 2 [file Image_2.tif]
