# Supplementary material for: Grey and harbor seals in France (mainland and Saint-Pierre et Miquelon): microbial communities and identification of a microbial source tracking seal marker
Source: Front Microbiol. 2024 Dec 4;15:1484094. doi: 10.3389/fmicb.2024.1484094 (PMC11652528; doi:10.3389/fmicb.2024.1484094)
Supplement: Supplementary file 4 [file Table_2.docx]

**Table S2.** qPCR target genes, primer/probe sequences and reaction conditions for MST candidates Seal_Bifido (Bifidobacteriaceae), *Fournierella*, *Atopobium, Slackia_1, Slackia_2,* and targeting seal feces.

| **Marker** | **Primers/probe** | **Sequences (5'-3')** | **Product size (bp)** | **Reaction conditions** | **Sensitivity (n of samples)/ specificity (n of samples)** |
| --- | --- | --- | --- | --- | --- |
| **Seal_Bifido (Bifidobacteriaceae)** | ***Seal_Bifido_F***  ***Seal_Bifido_R*** | AAAGTGAGAGTACCTCGC  CTCTACCGCACTCAAGTCAA | 191 | 1 cycle of 10 min at 95°C and 40 cycles of 15 sec 95°C and 1 min 60°C+ 1 dissociation step | Sensitivity of 89.8% (n=49 grey seals); specificity of 97.1% (n=69 non-target samples) |
| ***Fournierella*** | ***Fournierella_F***  ***Fournierella_R*** | CGGTACCTAAGAAGAAAGCCA  TCTGCACCACTCAAGAATAG | 183 | 1 cycle of 10 min at 95°C and 40 cycles of 15 sec 95°C and 1 min 60°C + 1 dissociation step | n. t. |
| ***Atopobium*** | ***Atopobium_F***  ***Atopobium_R*** | CAGCAGGGATGAGAAAAGACA  CTATGGTTTCAGAAGCGGCTC | 186 | 1 cycle of 10 min at 95°C and 40 cycles of 15 sec 95°C and 1 min 60°C+ 1 dissociation step | n. t. |
| ***Slackia_1*** | ***Slackia_F***  ***Slackia_1_R*** | TTCGGCAGGGAAGAATTTT  GACGGTGCCGCTTGAGGA | 146 | 1 cycle of 10 min at 95°C and 40 cycles of 15 sec 95°C and 1 min 60°C+ 1 dissociation step | n. t. |
| ***Slackia_2*** | ***Slackia_F***  ***Slackia_2_R*** | TTCGGCAGGGAAGAATTTT  CGCCTCAGCGTCAGTGCC | 302 | 1 cycle of 10 min at 95°C and 40 cycles of 15 sec 95°C and 1 min 60°C+ 1 dissociation step | n. t. |

n. t.: not tested; F: Forward; R: Reverse
